# The Implications of Relationships between Human Diseases and Metabolic Subpathways

**DOI:** 10.1371/journal.pone.0021131

**Published:** 2011-06-17

**Authors:** Xia Li, Chunquan Li, Desi Shang, Jing Li, Junwei Han, Yingbo Miao, Yan Wang, Qianghu Wang, Wei Li, Chao Wu, Yunpeng Zhang, Xiang Li, Qianlan Yao

**Affiliations:** 1 Bio-Pharmaceutical Key Laboratory of Heilongjiang Province, and College of Bioinformatics Science and Technology, Harbin Medical University, Harbin, China; 2 College of Pharmacy, Nankai University, Tianjin, China; Governmental Technical Research Centre of Finland, Finland

## Abstract

One of the challenging problems in the etiology of diseases is to explore the relationships between initiation and progression of diseases and abnormalities in local regions of metabolic pathways. To gain insight into such relationships, we applied the “k-clique” subpathway identification method to all disease-related gene sets. For each disease, the disease risk regions of metabolic pathways were then identified and considered as subpathways associated with the disease. We finally built a disease-metabolic subpathway network (DMSPN). Through analyses based on network biology, we found that a few subpathways, such as that of cytochrome P450, were highly connected with many diseases, and most belonged to fundamental metabolisms, suggesting that abnormalities of fundamental metabolic processes tend to cause more types of diseases. According to the categories of diseases and subpathways, we tested the clustering phenomenon of diseases and metabolic subpathways in the DMSPN. The results showed that both disease nodes and subpathway nodes displayed slight clustering phenomenon. We also tested correlations between network topology and genes within disease-related metabolic subpathways, and found that within a disease-related subpathway in the DMSPN, the ratio of disease genes and the ratio of tissue-specific genes significantly increased as the number of diseases caused by the subpathway increased. Surprisingly, the ratio of essential genes significantly decreased and the ratio of housekeeping genes remained relatively unchanged. Furthermore, the coexpression levels between disease genes and other types of genes were calculated for each subpathway in the DMSPN. The results indicated that those genes intensely influenced by disease genes, including essential genes and tissue-specific genes, might be significantly associated with the disease diversity of subpathways, suggesting that different kinds of genes within a disease-related subpathway may play significantly differential roles on the diversity of diseases caused by the corresponding subpathway.

## Introduction

Genes/proteins rarely function in isolation in and outside the cell, but interact with each other to form complex cellular pathways of metabolic, regulatory, or protein complexes to perform biological functions [1], [2], [3], [4]. The initiation and progression of diseases are highly associated with abnormalities in the biological functions of pathways. Especially, abnormalities in local regions (that is, subpathways) of cellular pathways contributes to the etiology of diseases [5], [6], [7], [8]. Among the cellular pathways, metabolic pathways are at the heart of the cell and are among most challenging biological networks and, arguably, the ones with most potential for immediate applicability [4], [5], [6]. Therefore, to elucidate the intricate relationships between human diseases and the disruption of underlying functional regions in metabolic pathways is an important challenge in the field of biology and medicine [5], [9], [10].

However, it is a challenge to identify disease-related regions of metabolic pathways. Some studies have successfully identified disease risk metabolic subpathways from low-throughput biological experimental studies. For example, an abnormality in norepinephrine metabolism, which is a sub-pathway of the ‘tyrosine metabolism’ pathway, was found to be associated with the initiation and progression of some diseases, such as cancer, and neurological, psychiatric, endocrine and cardiovascular diseases [8], [11]. If all disease-related subpathways can be identified by biological studies, the global relationships between metabolic subpathways and diseases could be constructed and studied in-depth from the viewpoint of network biology [3], which would can give global clues to possible metabolism-related treatment of diseases [3], [12], [13], [14], [15]. However, currently, it is difficult to construct the global relationships between metabolic subpathways and a variety of diseases via low-throughput biological experimental studies. Although disease-related genes can be identified effectively by a variety of methods [16], [17], the identification of disease-related metabolic subpathways is very difficult in terms of the lack of high-throughput experimental technology. High-throughput reactome arrays for the functional analysis of metabolic pathways were recently developed but have still not been applied to the identification of human disease-related subpathways [18].

Currently, the pathway-enriched methods are an efficient alterative strategy for establishing disease-pathway relationships by identifying disease risk pathways based on disease-related genes [8], [14], [19], [20], [21], [22], [23]. Information on disease-related genes, such as that available from the Genetic Association Database (GAD) [24] has become increasingly available for constructing high-quality disease–metabolic pathway relationships. Furthermore, the high quality pathway structure data available from the Kyoto Encyclopedia of Genes and Genomes (KEGG) [25] is invaluable for identifying the disease risk regions of pathways. Here, we constructed a global disease–metabolic subpathway network (DMSPN) in which nodes represent diseases or metabolic subpathways and these were connected by an edge if genes related to a particular disease were significantly enriched to the subpathway. We applied the “k-clique” pathway identification method to all disease-related gene sets. For each disease, the disease risk local regions of metabolic pathways were finally identified using disease genes and pathway structure data. These regions were considered as subpathways associated with the disease. We then used the methods of network biology to analyze global relationships between diseases and metabolic subpathways based on the basic properties of the DMSPN, the clustering phenomenon for disease nodes and subpathway nodes, the correlations between network topology and genes within disease-related metabolic subpathways. Through these analyses, our findings offer insight into the interplay between metabolic subpathways and human diseases.

## Results

### Construction of the disease–metabolic subpathway network

**Figure 1:**
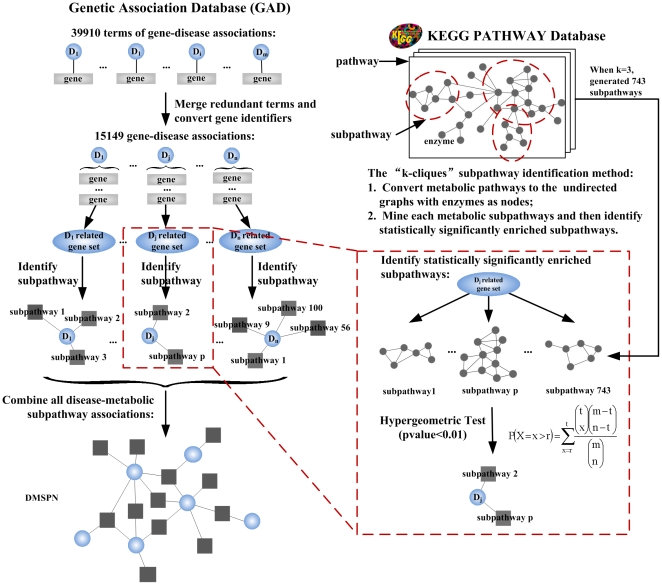
Schematic of the construction of the disease–metabolic subpathway network (DMSPN). We constructed a DMSPN based on disease genes and pathway structure data. First, we obtained 15149 unique gene–disease associations after processing all terms of gene–disease associations in the Genetic Association Database (GAD). Second, for each disease, we used the “k-cliques” subpathway identification method to identify statistically significantly enriched subpathways with P-value<0.01, to generate disease–metabolic subpathway associations for each disease. Finally, we combined these disease–metabolic subpathway associations to form the DMSPN.

**Figure 2:**
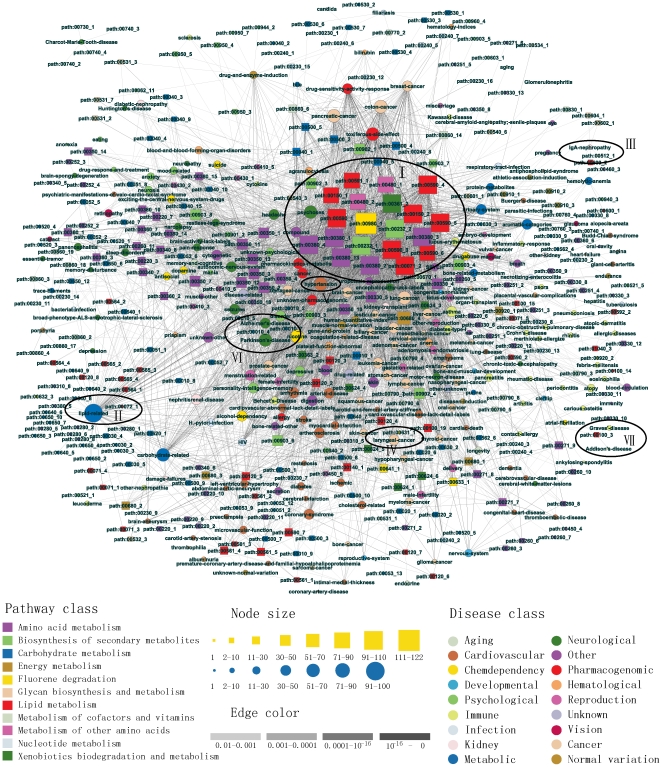
The DMSPN is a bipartite network. The circles and rectangles in the network correspond to diseases and metabolic subpathways respectively. A disease and a subpathway are connected by an edge if the set of disease genes are statistically significantly enriched to the corresponding subpathway. Node size is proportional to the degree of the node. Nodes are colored according to their categories, which include 18 disease classes from the Genetic Association Database (GAD) for disease nodes and 11 subpathway classes from the Kyoto Encyclopedia of Genes and Genomes (KEGG) pathway database for metabolic subpathway nodes. Edges are colored according to the enrichment significance (P-values) of associations between diseases and subpathways.

We constructed a disease–metabolic subpathway network based on disease genes and pathway structure data (Figure 1). First, we downloaded all terms of gene-disease associations (N = 39910) in the GAD (December 15, 2008). After some necessary dealing steps such as merging redundant terms and converting gene identifiers (see Text S1 and Dataset S1), 15149 unique gene–disease associations were finally obtained from 39910 primary terms. The 15149 gene–disease associations were composed of 412 diseases, which are naturally grouped by the GAD into 18 disease classes, and 2831 disease genes (see Dataset S2). Second, for each disease among the 412 diseases, we identified the disease-related subpathway based on the corresponding gene sets. We used the “k-cliques” subpathway identification method provided by the SubpathwayMiner software package [8] to identify statistically significantly enriched subpathways with a P-value<0.01 (see Text S1). This method is able to identify subpathways with a given distance parameter k, which means that the distance among all enzymes within the subpathways is no greater than k [8]. Briefly, each metabolic pathway is converted to an undirected graph with enzymes as nodes. After inputting the given disease-related genes and distance parameter k, the method can mine each metabolic subpathway and then identify statistically significantly enriched subpathways. In this study, we mainly discuss the DMSPN with k = 3. This parameter setting ensures that the identified disease risk subpathways are located in the disease-related local regions of pathways. When k = 3, a total of 743 metabolic subpathways were generated, of which 302 were significantly associated with 243 of the 412 diseases. These subpathways and diseases generated 4288 significant disease–metabolic subpathway associations. Finally, we combined these disease–metabolic subpathway associations to construct the DMSPN with k = 3 (see Dataset S3). The network was a bipartite network consisting of two disjointed sets of nodes. One set corresponded to 243 diseases, and the other set corresponded to 302 disease-related metabolic subpathways (circular nodes represent diseases and rectangular nodes represent subpathways in Figure 2). A disease and a subpathway were connected if the corresponding disease-related genes were significantly enriched to the corresponding subpathway. We also developed a platform for constructing disease–metabolic subpathway networks (Figure S1), which was integrated into the SubpathwayMiner package freely available at http://cran.r-project.org/web/packages/SubpathwayMiner/. Using this platform, we constructed disease–metabolic subpathway networks based on the different scales of subpathways.

### The basic network features of the DMSPN

**Figure 3:**
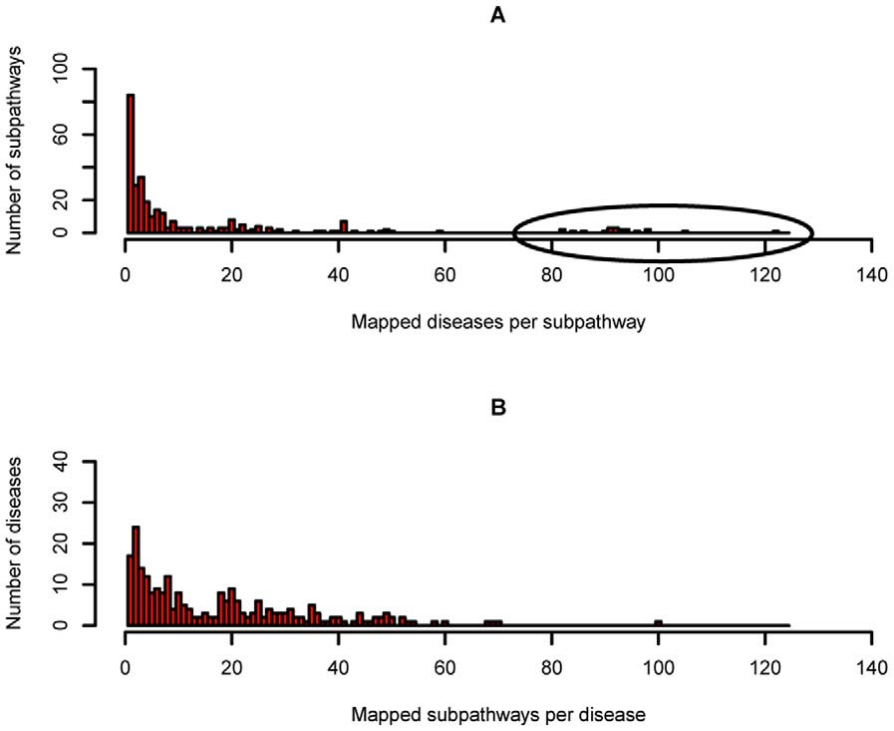
Mapping between diseases and subpathways. (**A**) Distribution of the number of mapped diseases per subpathway. (**B**) Distribution of the number of mapped subpathways per disease.

The DMSPN with k = 3 was composed of 545 nodes (302 subpathways and 243 diseases), and 4288 edges (Figure 2). The edges in the DMSPN were denser than expected by chance with P-values<0.001 (see Text S1 and Figure S2C), and most nodes formed a giant connected component, suggesting that the diseases and metabolic subpathways were much closely connected at the system level. The degree of subpathway nodes as well as that of disease nodes (circular and rectangular nodes of different sizes in Figure 2) had fat tails (Figure 3). On average, a subpathway in the DMSPN was associated with 14 diseases, and the initiation and progression of a disease involved 20 subpathways. The degree distribution of subpathway nodes was much broader than that of disease nodes. To further test the differences between the degree distribution of the subpathway and disease nodes, we generated 1000 random networks and compared them with the actual DMSPN (see Text S1). We found that the degree distribution of the subpathway nodes of the actual DMSPN was significantly broader than that of the random networks (P-value<10e–10, see SI Figure S2A), whereas the distribution of disease nodes did not display such a highly significant difference (P-value = 0.02, see SI Figure S2B), suggesting that the different subpathways in the DMSPN possessed the considerable differences with respect to causing diseases. On the one hand, few metabolic subpathways were linked to many diseases. The degrees of only 20 subpathways (nodes with degree>80 in Figure 3A, and ellipse I region at the central top part of Figure 2) among the 302 total subpathways were significantly higher than the degrees of other subpathways. Interestingly, most of these connected subpathways were involved with lipid metabolism (9/20) (red rectangular nodes) and amino acid metabolism (7/20) (orchid rectangular nodes), suggesting that abnormalities in fundamental metabolism may tend to cause more types of disease. For example, for lipid metabolism, five of the nine subpathways were involved with arachidonic acid metabolism. Some studies showed that arachidonic acid could generate many compounds with different cellular or extracellular tissue and organ targets, causing a wide range of clinical effects [26]. Arachidonic acid metabolism has been reported to be associated with different kinds of diseases, including inflammation, hypertension, diabetes, cardiovascular diseases, depression, schizophrenia, Alzheimer's disease and cancer [27]. Of seven amino acid metabolism subpathways, five subpathways belonged to tryptophan metabolism. The dysfunction of tryptophan metabolism has been found to be associated with many diseases including cancer, and psychological, immune and cardiovascular diseases [28], [29], [30], [31]. Of the other 4 of the 20 subpathways with degree>80, the subpathways path:00980_1 and path:00361_1 with degrees of 122 and 105, respectively, belonged to metabolism of xenobiotics by cytochrome P450 and gamma-hexachlorocyclohexane degradation, which were directly related to xenobiotic degradation and metabolism. Cytochrome P450 is involved in the bioactivation and detoxification of a variety of xenobiotics [32]. Abnormal metabolism of xenobiotics by P450 may cause inborn errors in metabolism and contribute to many clinically relevant diseases [33]. The subpathways path:00232_1 and path:00232_2 (light green rectangular nodes) belonged to caffeine metabolism, were associated with 98 diseases in the DMSPN and had been proved to be potentially related to many diseases including Alzheimer's disease, asthma, cancer, diabetes and Parkinson's disease [34]. On the other hand, many metabolic subpathways were connected to only a few diseases in the DMSPN. For example, path:00072_1, path:00512_1 and path:00631_1 were only connected to one disease. The synthesis and degradation pathway of ketone bodies (path:00072_1), which belonged to a subpathway of lipid metabolism, was only associated with lipid-related diseases (ellipse II in Figure 2). Similarly, the O-glycan biosynthesis subpathway (path:00512_1) was only linked to IgA-nephropathy in the DMSPN (ellipse III) [35], and the 1,2-dichloroethane degradation subpathway (path:00631_1), which was found to be associated with the initiation and progression of cancer [36], was only linked to laryngeal cancer (ellipse IV).

Although the degree distribution of disease nodes was no broader than that of the subpathway nodes, the degree of diseases also spanned a wide range from 1 to 100 (Figure 3B, and circular nodes of different sizes in Figure 2). Diseases with high degrees displayed significantly different characteristics compared with diseases with low degrees. Most diseases with high degrees were well-known, highly heterogeneous, complex diseases such as Parkinson's disease (ellipse V), hypertension (ellipse VI), Alzheimer's disease (ellipse VI) and some cancers (the central part of Figure 2). By contrast, some rare and lowly complex diseases were usually only associated with a few subpathways. Interestingly, many diseases with low degrees, including Addison's disease (ellipse VII) and Graves' disease (ellipse VII), belonged to autoimmune diseases (khaki circular nodes at the lower right part of Figure 2). Autoimmune diseases occur when the body's immune system attacks its own special tissues or systems, thinking they are foreign threats. Therefore, the initiation and progression of these diseases are usually pathway-specific. For example, Addison's disease, which was only associated with the subpathway path:00100_3 (a subpathway of the steroid biosynthesis pathway) in the DMSPN, is mainly caused by impaired steroidogenesis [37].

### Evaluating the clustering phenomenon of diseases and subpathways

As illustrated in Figure 2, diseases were divided into 18 classes (circular nodes of different colors) according to the GAD, and subpathways were divided into 11 classes (rectangular nodes of different colors) according to the KEGG. If a subpathway node has a high degree, the subpathway is associated with the initiation and progression of more diseases. However, we questioned whether these potential diseases induced by the subpathway belong to more different disease classes. To test this, we calculated the disease diversity of each subpathway in the DMSPN. Disease diversity of a subpathway is defined as the unique class numbers of diseases associated with the subpathway divided by the total numbers of disease classes. We found that there was a significant positive correlation between disease diversity and degree of a subpathway (P-value<2.2e–16, see SI Figure S3), suggesting that the higher the subpathway degree, the more types of disease are caused by disruption of the subpathway.

**Figure 4:**
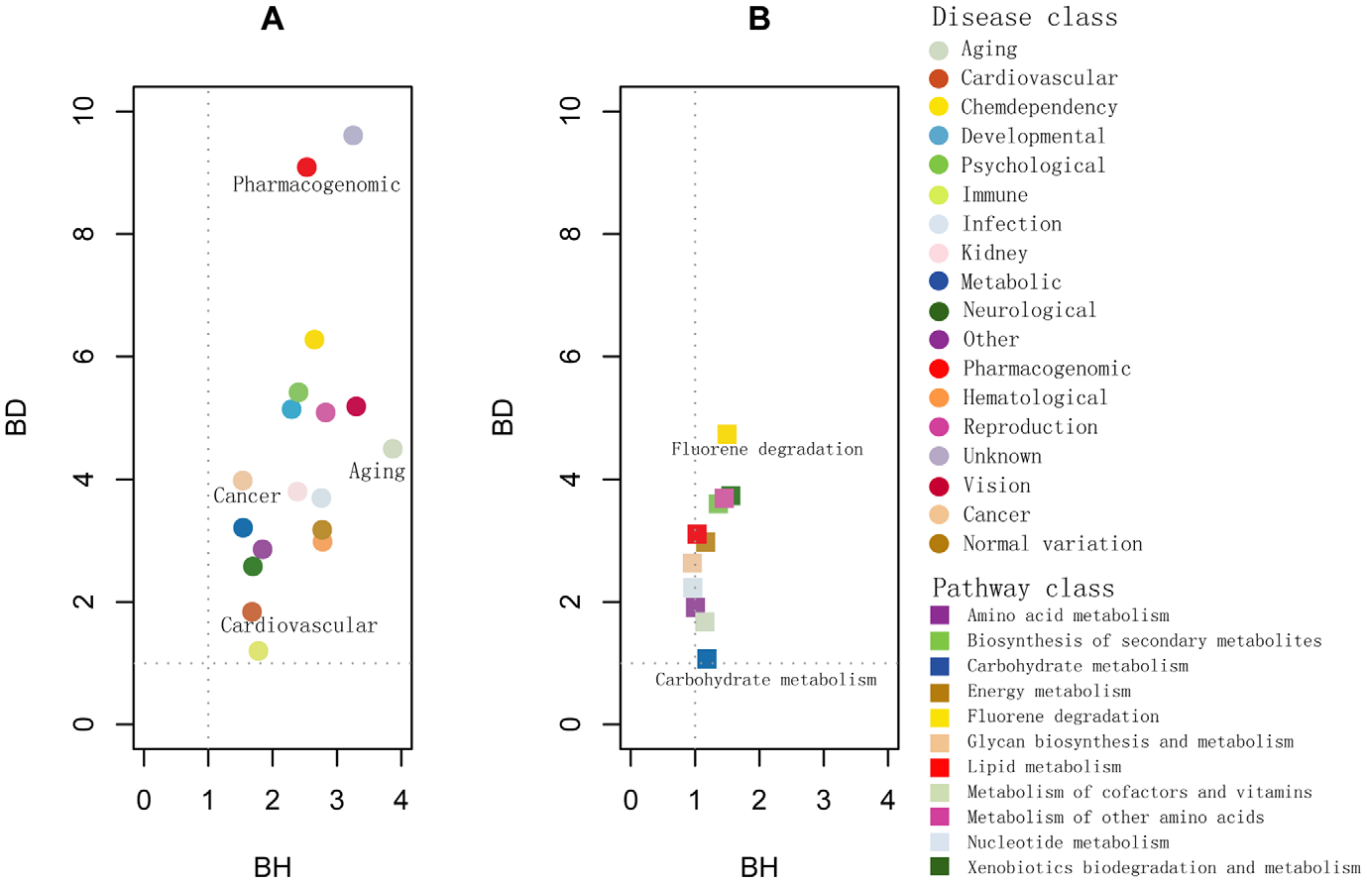
Evaluating the clustering phenomenon using BD and BH measures. (**A**) The plot of BD and BH of 18 disease classes. (**B**) The plot of BD and BH of 11 metabolic subpathway classes.

Some studies have shown that diseases within the same disease class tend to be clustered into densely connected groups in disease networks [12], [14], [38], [39]. To probe whether functional clustering of DMSPN exists, we calculated the (BD, BH) values of each class to test the clustering phenomenon of disease classes and subpathway classes in the DMSPN respectively (see Materials and methods). Briefly, for a disease class, BD>1 (BD<1) indicates that diseases in the disease class tend to connect more (less) densely among themselves than the random expectation. BH>1 (BH<1) means that diseases in the disease class have more (fewer) connections to diseases in other classes than the random expectation. If BD>BH, diseases in the disease class tend to display a clustering phenomenon in the DMSPN. If BD>1 and BH<1, diseases in the disease class tend to display a clear clustering phenomenon in the DMSPN. Similarly, we can also calculate the BD and BH values of subpathways in a subpathway class. The results showed that for disease classes, the average values of (BD, BH) were 4.4 and 2.3 respectively (Figure 4A). The average value of BD was one times more than that of BH, suggesting that diseases in the same disease class tend to connect more densely than expected by chance. However, these diseases still connected densely with diseases in other disease classes as demonstrated by their high BH value (BH>1). For example, the BD value of “pharmacogenomic” class was 3 times more than the BH value that was equal to 2.5 (Figure 4A), suggesting that diseases in the class have more similar mechanism of happen, but have still similar mechanism of happen with some diseases in other disease classes. Therefore, diseases in the same disease class displayed a slight clustering tendency. However, for several disease classes, including the aging disease class and the cardiovascular disease class, the BD values were similar to the BH values. For subpathway classes, the average values of BD and BH were 2.8 and 1.2 respectively (Figure 4B). The average value of BD was one times more than that of BH, suggesting that subpathways in the same subpathway class display a slight clustering tendency. For example, fluorene degradation (BD = 4.7, BH = 1.4), nucleotide metabolism (BD = 2.2, BH = 0.9) and glycan biosynthesis (BD = 2.6, BH = 0.9) displayed a slight clustering tendency in the DMSPN, suggesting that the disruption of these subpathways tends to cause similar diseases. However, carbohydrate metabolism (BD = 1.0, BH = 1.1) and amino acid metabolism (BD = 1.9, BH = 1.0) displayed low value.

**Figure 5:**
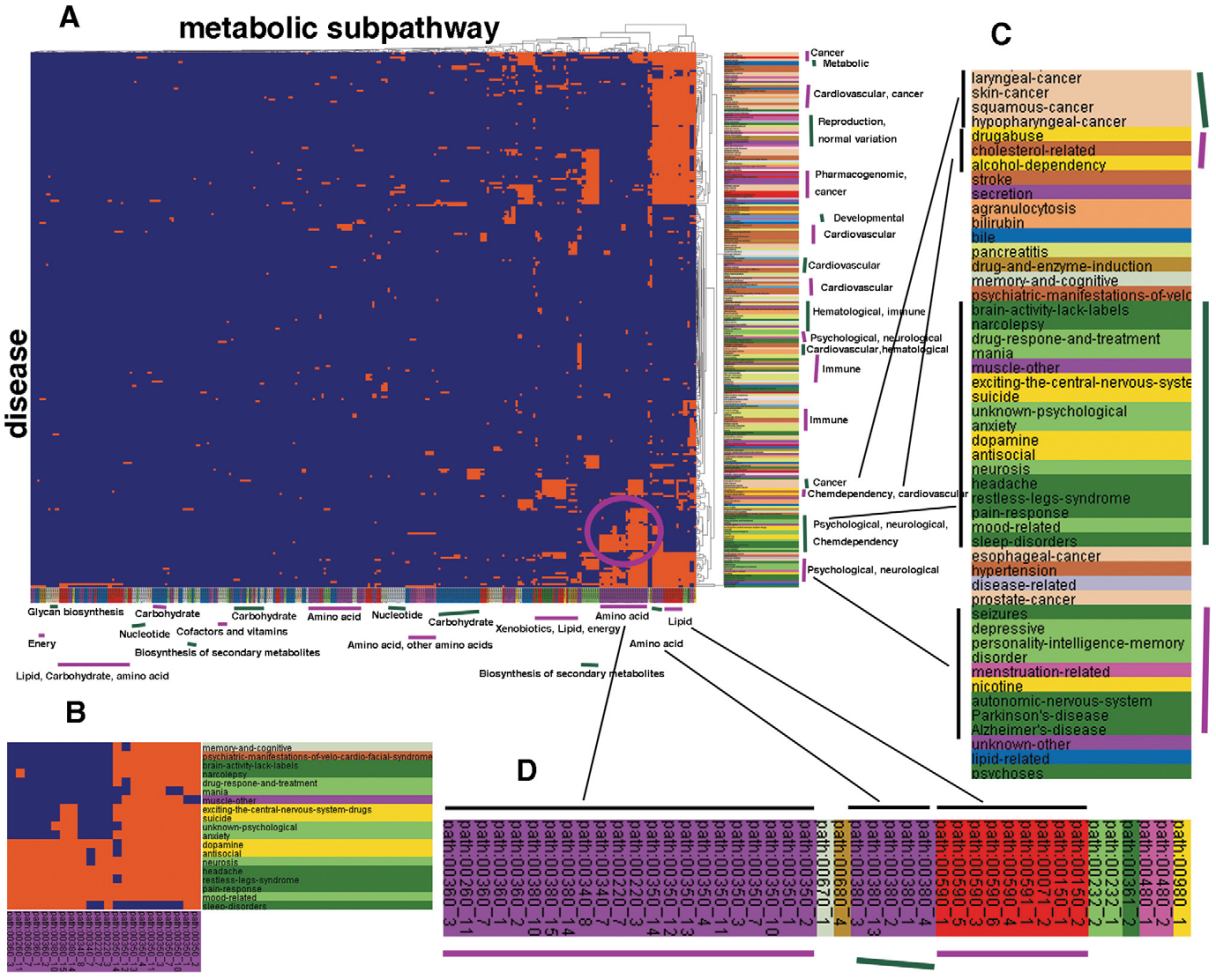
Hierarchical clustering on the DMSPN using the city-block distance and complete linkage method in the Cluster3 software package and JavaTreeView imaging software. (**A**) Hierarchical clustering between 243 diseases and 302 metabolic subpathways. The corresponding cell was colored orange if there was an edge between the disease and subpathway in the DMSPN. Disease labels and subpathway labels were colored according to the disease class colors and subpathway class colors used in Figure 2 and 3. (**B**) Zoom-in plot of part of the pink circled region in Figure 5A, showing the psychological, neurological, and chemdependency disease classes and amino acid metabolism subpathways. (**C**) Zoom-in plot of part of the disease labels in the lower-right region of Figure 5A. (**D**) Zoom-in plot of part of the subpathway labels in the lower-right region of Figure 5A.

The (BD, BH) measure evaluated clustering tendency for a given class and showed that diseases (and subpathways) in the same disease (and subpathway) class displayed a slight clustering tendency. After clustering the nodes in the DMSPN, without considering disease class information, diseases in the same clusters should tend to belong to the same disease class. To detect this, we performed hierarchical clustering [40], [41] on the DMSPN (Figure 5A). The results showed that diseases belonging to the same disease class tend to group together. However, this does not mean that all diseases in the same disease class were perfectly clustered. In fact, diseases in the same class tend to be clustered at the local level, but not at the global level (e.g., the neurological and psychological diseases (dark and light green labels in Figure 5C)). That is, diseases in the same disease class tended to form many small clusters instead of individual large clusters. This phenomenon was also interpreted by the high BD and BH values of the disease class described above, because the high BH value indicates that some diseases in a disease class are closely related to diseases in other classes. Next, we questioned what factors contribute to the high degree of similarity between diseases belonging to different disease classes. One factor may be the degree of genetic similarity in a disease class. Then, what contribute to the high similarity of diseases belonging to different disease classes? For example, the neurological and psychological diseases have similar genetic origins and their genetic similarity of them may make the two kinds of diseases merge into one large cluster when clustering them (dark and light green labels in Figure 5C). Another factor may be different viewpoints with regard to classifying a disease, which divide some the co-characteristic diseases into different disease classes and cause the ambiguous boundaries between different diseases. For example, although the “chemdependency” diseases are obviously different from the neurological and psychological diseases, some diseases in the “chemdependency” class, including nicotine, dopamine and antisocial, not only relate to chemdependency but also to neurology and psychology. In the DMSPN, these diseases were clustered into the neurological and psychological cluster at the global level (Figure 5C). In addition, the high correlations between metabolic subpathways also decreased the clustering phenomenon of the DMSPN.

### Correlations between network topology and genes within disease-related metabolic subpathways

**Figure 6:**
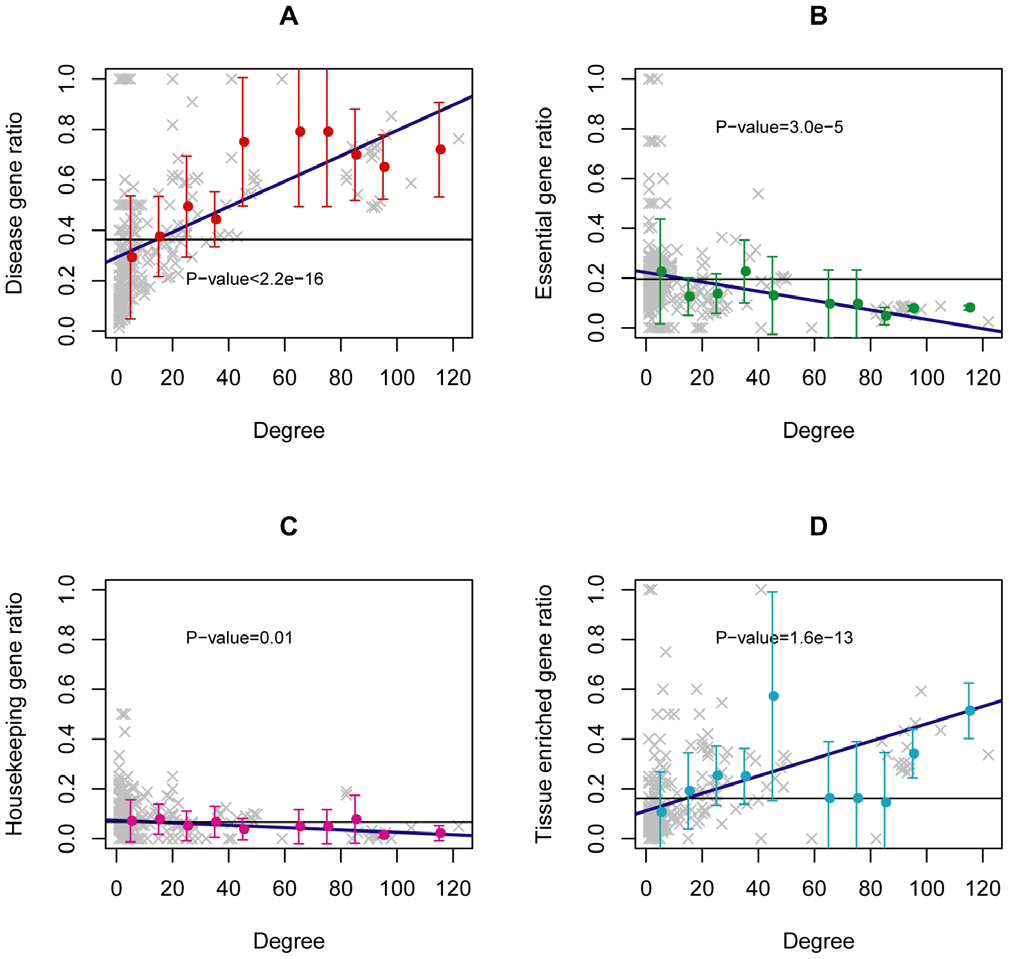
Correlations between network topology and genes within disease-related metabolic subpathways. For each subpathway in the DMSPN, the degree of the subpathway and the ratio of different types of genes within the subpathway were calculated. Gray “×” symbols represent subpathways. Black horizontal lines are the average of the ratio of genes. Color points correspond to the binned ratio values and error bars correspond to the standard deviations of the binned ratio values. The linear regression model was used to test the trends in correlations and the significance of these trends was estimated. (**A**) The ratio of disease genes divided by all genes within the subpathways. (**B**) The ratio of essential genes divided by all genes in the subpathways. (**C**) The ratio of housekeeping genes divided by all genes in the subpathways. (**D**) The ratio of tissue-specific genes divided by all genes in the subpathways.

Many diseases, especially complex diseases, are usually related to disruption of underlying functions in metabolic subpathways. Most of the biological functions of a metabolic subpathway are carried out by biochemical interactions between gene products within subpathways. Therefore, involvement of a high proportion of disease genes in a subpathway might increase the possibility of disruption of the corresponding subpathway, finally leading to more diseases. To examine this, we measured the ratio of the number of disease genes to the number of total genes within each subpathway of the DMSPN. We found that the ratio significantly increased as the degree of a subpathway increased (P-value<2.2e–16; Figure 6A). Moreover, there were on average 20 disease genes in a disease-related subpathway, representing 36.3% of the total genes in a subpathway. These suggested that subpathways associated with more diseases tend to contain higher ratios of disease genes. Similarly, metabolic subpathway regions with a high ratio of disease genes also tend to cause more types of diseases due to the high positive correlation between degree and disease diversity of the subpathway node (see Figure S3).

The ratio of disease genes within a metabolic subpathway was significantly associated with the number of diseases induced by dysfunction of the corresponding subpathway. Other types of genes within these disease risk regions could therefore also be significantly associated with disease diversity of subpathways. To examine this, we firstly tested essential genes, whose functions are necessary for organisms to survive and reproduce. Essential genes are often compared with disease genes in many studies. Some recent studies have found that diseases genes and essential genes may encode hubs in protein interaction network [42], [43], [44]. Other studies reported only a weak correlation between disease genes and hubs [45]. Essential genes may have a direct association with cancer [12] and may be regarded as the most severe “disease” genes [46]. Can the disease-related functional differences between disease genes and essential genes be observed in the subpathway regions of the DMSPN? To address this question and test relationships between essential genes and the topology of the DMSPN, we measured the ratio of essential genes within each subpathway in the DMSPN. 2486 human essential genes were obtained according to the lethal phenotype information of the corresponding mouse orthology (see Materials and methods) from the Mouse Genome informatics [47], [48]. We then matched them to each subpathway for calculating the ratio of essential genes of subpathways. Surprisingly, the results showed that the ratio of essential genes to the number of total genes within each subpathway decreased significantly as the degree of the corresponding subpathway increased (P-value<2.2e–16) (Figure 6B), which was a significant negative correlation between the ratio of essential genes and the degree of a subpathway. The trend between essential genes and the topology of the DMSPN was opposite to the trend between disease genes and the topology of the DMSPN, suggesting that the DMSPN is able to distinguish well the disease-related functional differences between disease genes and essential genes. This result also indicated that regions of metabolic pathways with a high ratio of essential genes were less likely to lead to more types of diseases. Mutation of essential genes usually causes severe functional impairment that tends to result in lethality. Thus, abnormalities within a subpathway that contains a higher ratio of essential genes might lead to lethality in patients before suffering from more diseases whereas abnormality of the subpathway that contains higher ratio of disease genes more easily make patients survive or die after suffering from more and more diseases.

We further tested housekeeping (HK) genes and tissue-specific genes (TS). HK genes are constitutively expressed in all tissues and cell types to maintain basic cellular functions. Whereas, TS genes are expressed at a much higher level in a single tissue than in others. Expression regulation differences between HK and TS genes were analyzed recently [49], [50], [51]. We downloaded the HK and TS gene dataset used by She et al. [49] which contained 1522 human HK genes and 975 TS genes identified from the gene expression profiles of 42 normal human tissues using high-density microarrays and the conservative identification criteria. We found that there were the higher ratio of TS genes as a subpathway was associated with more diseases (P-value<1.63e–13) (Figure 6D). However, there was no significant correlation between the ratio of HK genes and the degree of subpathways (P-value = 0.01) (Figure 6C). Many studies have shown that HK genes are less likely to mutate and are more conserved than TS genes, that they are more stably expressed and that they are more enriched in basic maintenance biological processes of the cell. HK genes may thus play a basic role in the cell and maintain stable numbers regardless of whether abnormalities of the subpathway regions that contain these genes lead to more types of diseases. However, TS genes show similar trends with disease genes, for disease genes tend to be expressed specifically in some tissues. In summary, the DMSPN is able to distinguish well the disease-related functional difference between HK genes and TS genes.

**Figure 7:**
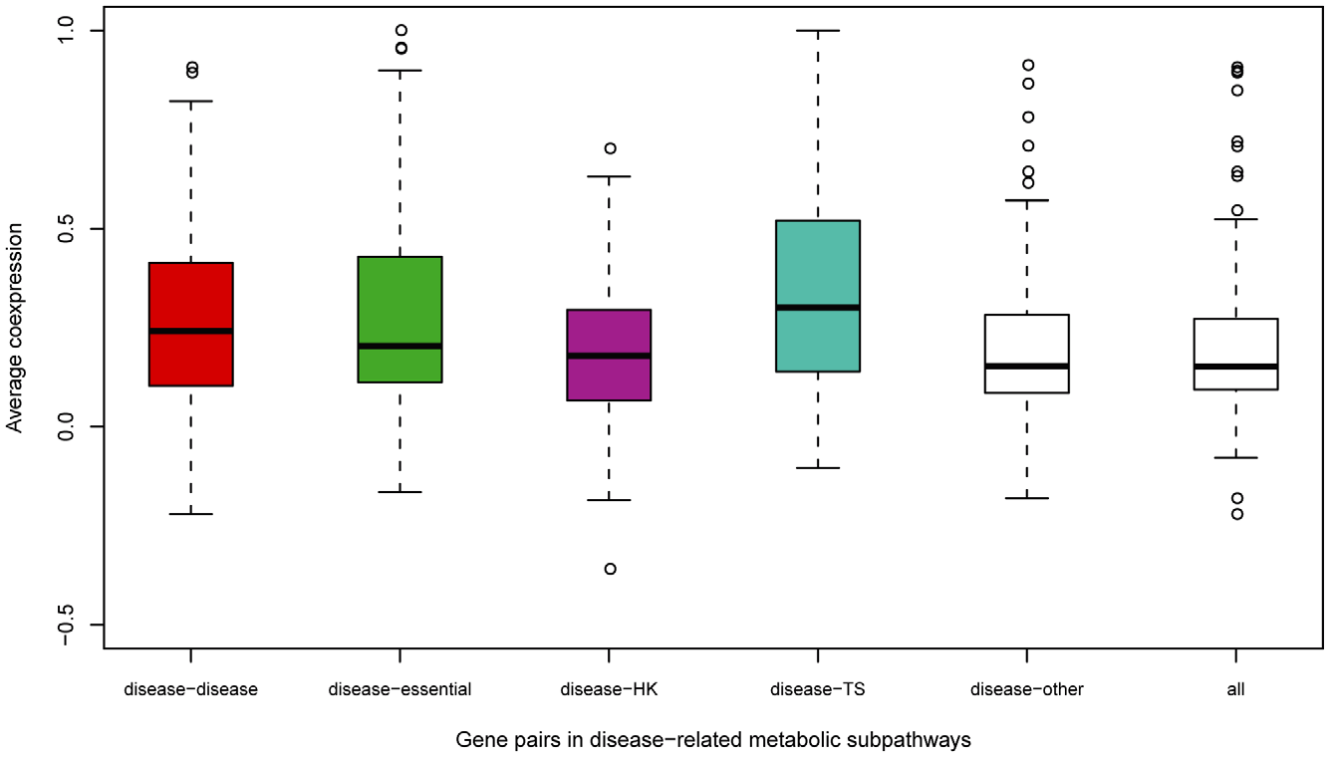
The average coexpression of gene pairs within the disease-related metabolic subpathways.

Disease genes within the potential disease-related subpathways of DMSPN may play key roles in regulating these subpathways. Due to the differences of trend between ratio of different types of genes and degree of subpathways, disease genes should adopt different regulation intensity and have different coexpression with other types of genes at expression levels. To address this issue, we computed coexpression between genes using expression data from 36 different human tissue microarray experiments [52]. The results showed that within subpathways, the median values for the average coexpression between: disease genes and disease genes, disease genes and essential genes, disease genes and HK genes, disease genes and TS genes, disease genes and other genes, and between all genes, were 0.24, 0.20, 0.17, 0.30, 0.15 and 0.15 respectively. As illustrated in Figure 7, disease genes within a subpathway have higher average coexpression values than expected by random (P-value<0.0007, Wilcoxon rank-sum test, we used the average coexpression between all genes as a random control). Moreover, disease genes showed higher coexpression with essential genes compared with that expected by chance (P-value<3.907e–05, Wilcoxon rank-sum test). However, coexpression between disease genes and HK genes did not show significant differences compared with that expected by chance (p-value = 0.68). These results suggest that disease genes may weakly influence HK genes, but significantly influence essential genes. The different regulation intensities might explain the differences of trend between the ratio of different types of genes and the degree of subpathways. As a result, those genes intensely influenced by disease genes might be closely associated with disease diversity of subpathways. Taken together, these findings indicated that the diversity of diseases caused by abnormalities in a subpathway region might be significantly associated with disease genes, essential genes and TS genes within the corresponding subpathway. However, significant correlations between HK genes and disease diversity of subpathways may not exist.

## Discussion

We constructed a disease–metabolic subpathway network using the disease-gene associations and “k-clique” subpathway identification method. We used the “k-clique” method to divide large pathways into multiple subpathways. When k = 3, the distance among all enzymes within a subpathway was no greater than 3. This ensured that the identified disease risk subpathways were located in the disease-related local regions of pathways, and also increased the tendency for genes in a subpathway to share similar biological functions and be involved in similar biological processes. To date, most pathway identification methods have only identified disease-related entire pathways without considering the scale of these pathways. However, each pathway has the obviously different scales. These differences in scale hinder the evaluation of disease-related pathways from global networks and the identification of disease-related high risk subpathways that are usually located in the local regions of pathways. Through employing the subpathway identification method, we were able to ensure that each subpathway was represented on a small scale. Moreover, the identified subpathways could be located within the disease risk regions of these pathways. By applying this methodology to all diseases, we were finally able to investigate global relationships between diseases and their corresponding regions of pathways.

Through our analyses of the DMSPN based on network biology, we found that the degree distribution of subpathway nodes of the actual DMSPN was significantly broader than that of random networks. A few subpathways were significantly linked to many diseases. However, most of these metabolic subpathways with high degrees belonged to fundamental metabolism pathways, suggesting that abnormalities in fundamental metabolism processes may cause more types of diseases. To test the clustering phenomenon of diseases and metabolic subpathways, we examined the (BD, BH) values based on both disease and subpathway class information from the GAD and KEGG databases. We also performed hierarchical clustering on the DMSPN without considering disease class information. The results showed that disease and subpathway classes displayed the slight clustering. For many classes, members within them still maintained many links with members of other classes although there were more links between members within the class. Therefore, diseases in the same disease class tended to form many small clusters instead of individual large clusters. Furthermore, we tested correlations between network topology and genes within disease-related metabolic subpathways. We found that the ratio of disease genes and the ratio of tissue-specific genes in a subpathway significantly increased as the degree of the subpathway increasesd. However, the ratio of essential genes significantly reduces and the ratio of housekeeping genes remained relatively unchanged. Furthermore, the coexpression levels between disease genes and other types of genes were calculated for each subpathway in the DMSPN. The results indicated that those genes intensely influenced by disease genes, including essential genes and tissue-specific genes, might be significantly associated with the disease diversity of subpathways. Taken together, these results provided strong support for the functional importance of the DMSPN.

To confirm the validity of our results, we also constructed the DMSPN with k = 4 and repeated the analyses. We find that the results reported in this paper were similar to the results of the DMSPN with k = 4 (see Text S1, Figure S4 and S5). We also noted that the completeness of the DMSPN is limited by the completeness of metabolic pathway data, disease–gene associations, and the false positive results obtained by enrichment analysis method. Improvements in the quality of this input data will provide a more accurate and robust DMSPN. Although these data sets and methodology are far from complete, our network analyses still provide statistically significant characteristics of the relationships between diseases and metabolic subpathways.

## Materials and Methods

### The Genetic Association Database (GAD)

To predict disease–metabolic subpathway associations, we first obtained disease–gene associations from the GAD. The GAD is the NIH-supported public repository for human genetic association studies of complex diseases, which contains the complete known gene–phenotype associations and include non-Mendelian common complex diseases [24]. We downloaded all gene-phenotype relationships (N = 39910) in the GAD (December 15, 2008) and processed this data by merging redundant terms (e.g., 112 “alcohol abuse” terms were merged). The detailed methodology used for processing this data was provided in Text S1. Finally, we obtained 15149 unique gene–disease relationships from 39910 primary terms. These associations were composed of 412 diseases and 2832 disease genes (see Dataset S2). We can also obtain the information of disease classes from the GAD. In particular, each disease corresponds to a disease class and in total 412 diseases belonged to 18 disease classes.

### Essential genes

To obtain human essential genes, we used the phenotype information of the corresponding mouse orthology. If a mouse suffered from the lethality when a particular gene was knocked out, a human ortholog of this gene was defined as an essential gene. This type of methodology has been widely applied to obtain information on human essential genes [12], [13]. Both human–mouse orthology data and mouse phenotype data were obtained from the Mouse Genome informatics [47], [48] (August 6, 2009). For the phenotype data, the embryonic, prenatal and postnatal lethality were treated as lethal phenotypes. Finally, 2486 mouse-lethal human orthologs were classified as human essential genes.

### Housekeeping (HK) genes and tissue-specific (TS) genes

HK genes are constitutively expressed in all tissues to maintain cellular functions, while TS genes are expressed at a much higher level in a single tissue than in others. Expression regulation differences between HK and TS genes were analyzed recently by She et al. [49]. We downloaded the HK and TS genes dataset which was used in that paper. The dataset contains 1522 HK genes and 975 TS genes, which were identified from the gene expression profiles of 42 normal human tissues using high-density microarrays and the conservative identification criteria [49].

### Computation of BD and BH

Park et al. developed D and H measures to capture the detailed interplay between the network structure and node properties when nodes in a network belong to two distinct classes [53]. These measures have been successfully applied to the modularity evaluation of protein–protein interaction networks [53] and disease networks [38]. In this study, we revised the D and H measures for one-mode networks (e.g., protein–protein interaction networks) to BD and BH measures for bipartite networks, such as the disease–metabolic subpathway network (see Text S1). For diseases in each disease class in the DMSPN, BD and BH were calculated and finally 18 pairs of (BD, BH) values are shown in Figure 4A. BD>1 (BD<1) indicate that diseases in the disease class tend to connect more (less) densely among themselves than expected by chance. Similarly, BH>1 (BH<1) means that diseases in the disease class have more (fewer) connections to diseases in other classes than the random expectation. If BD>BH, diseases in the disease class tend to display a clustering phenomenon in the DMSPN. If BD>1 and BH<1, diseases in the disease class tend to display a clear clustering phenomenon in the DMSPN. Similarly, we are also able to calculate the BD and BH values of subpathways in a subpathway class (Figure 4B).

## Supporting Information

Figure S1 **Screenshot of using the subpathway identification method provided in the SubpathwayMiner package to construct the disease–metabolic subpathway network.** After installing the SubpathwayMiner package in R, we can use the generateNetwork function to construct the disease–metabolic subpathway network with the different distance parameter k.
(TIF)

Figure S2 **The basic network features of the DMSPN.** (A) The degree distribution of subpathway nodes of the actual DMSPN was significantly broader than that of random networks (P-value<10e-10). (B) The degree distribution of disease node did not display such highly significant difference (P-value = 0.02). (C) The edges in the DMSPN were significantly denser than expected by chance (P-value<0.001). (D) The size of giant component of the DMSPN was significantly smaller than expected by chance (P-value<0.001).
(TIF)

Figure S3 **Correlation between the disease diversity and the degree within disease-related metabolic subpathways.** For each subpathway in the DMSPN, the degree of subpathway and the disease diversity were calculated. Gray “×” symbols represented subpathways. Black horizontal lines are the average of value of disease diversity. Color points correspond to the binned ratio values and error bars correspond to the standard deviations of the binned ratio values. The linear regression model was used to test the trends in correlations and the significance of these trends was estimated. The result showed that there was a significant positive correlation between the disease diversity and the degree of a subpathway (P-value<2.2e–16).
(TIF)

Figure S4 **Correlations between network topology and genes within disease-related metabolic subpathways.** For each subpathway in the DMSPN with k = 4, the degree of subpathway and the ratio of different types of genes within subpathway were calculated. Gray “×” symbols represented subpathways. Black horizontal lines are the average of the ratio of genes. Color points correspond to the binned ratio values and error bars correspond to the standard deviations of the binned ratio values. The linear regression model was used to test the trends in correlations and the significance of these trends was estimated. (**A**) The ratio of disease genes divided by all genes within the subpathways. (**B**) The ratio of essential genes divided by all genes in the subpathways. (**C**) The ratio of housekeeping genes divided by all genes in the subpathways. (**D**) The ratio of tissue-specific genes divided by all genes in subpathways.
(TIF)

Figure S5 **The average coexpression of gene pairs within disease-related metabolic subpathways in the DMSPN with k = 4.**
(TIF)

Dataset S1 **Examples of merging broad phenotypes.**
(XLS)

Dataset S2 **The curated gene–disease associations from the GAD.**
(XLS)

Dataset S3 **The disease-metabolic subpathway associations.**
(XLS)

Text S1 **Supplementary information text.**
(DOC)
