# Supplementary figures and images for: The Implications of Relationships between Human Diseases and Metabolic Subpathways
Source: PLoS One. 2011 Jun 17;6(6):e21131. doi: 10.1371/journal.pone.0021131 (PMC3117879; doi:10.1371/journal.pone.0021131)

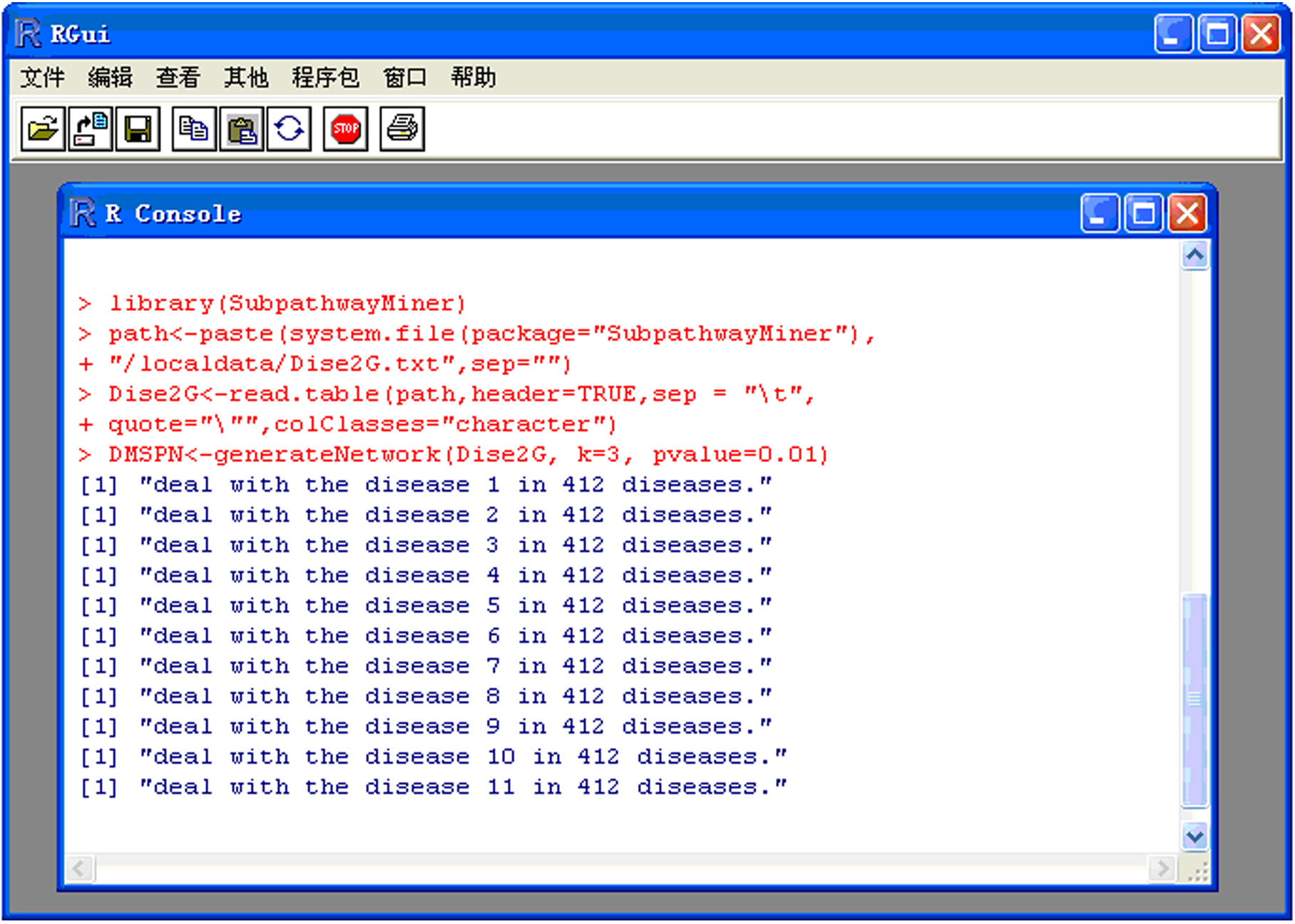

Supplement: Figure S1 — Screenshot of using the subpathway identification method provided in the SubpathwayMiner package to construct the disease–metabolic subpathway network. After installing the SubpathwayMiner package in R, we can use the generateNetwork function to construct the disease–metabolic subpathway network with the different distance parameter k. (TIF) [file pone.0021131.s001.tif]

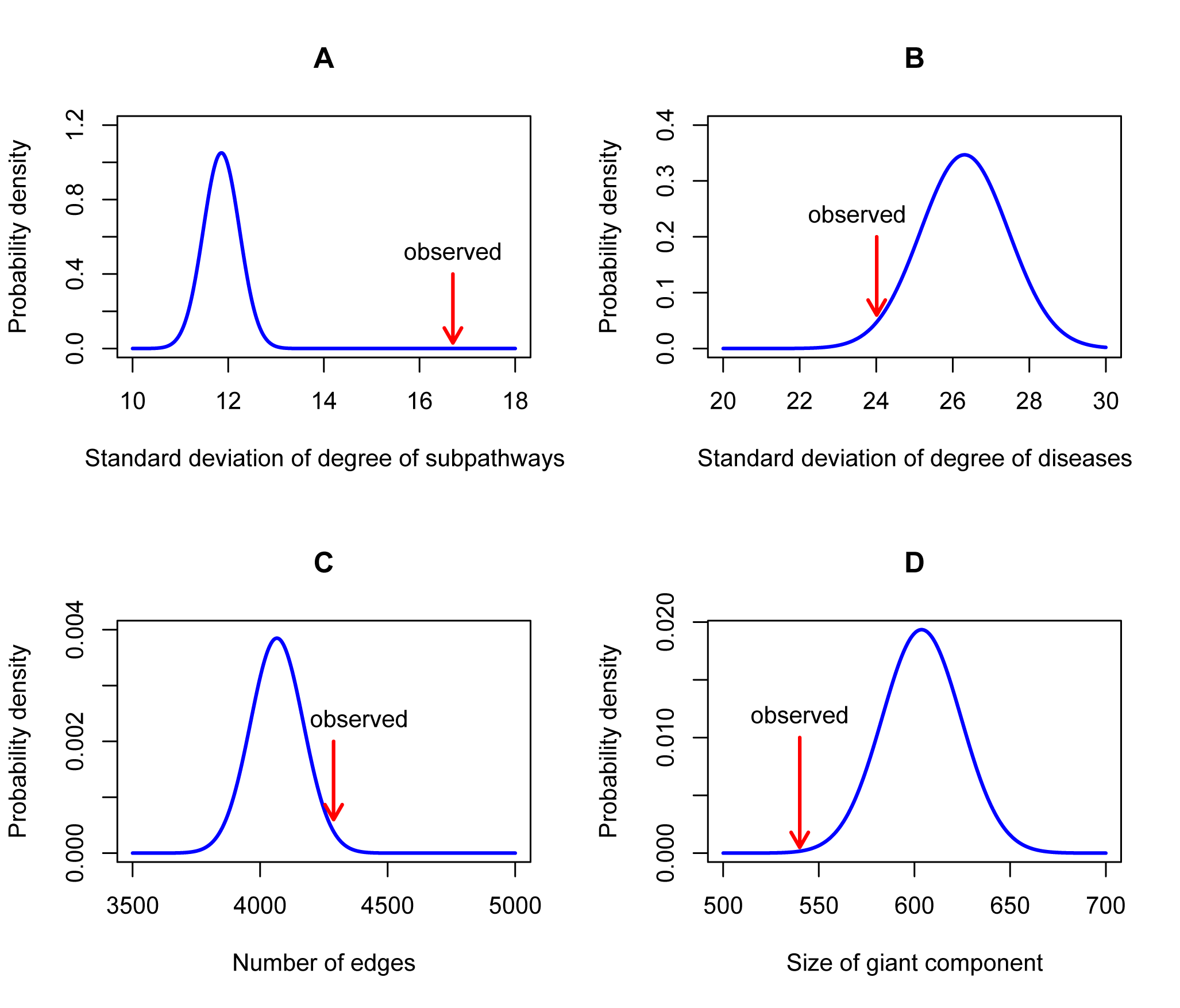

Supplement: Figure S2 — The basic network features of the DMSPN. (A) The degree distribution of subpathway nodes of the actual DMSPN was significantly broader than that of random networks (P-value<10e-10). (B) The degree distribution of disease node did not display such highly significant difference (P-value = 0.02). (C) The edges in the DMSPN were significantly denser than expected by chance (P-value<0.001). (D) The size of giant component of the DMSPN was significantly smaller than expected by chance (P-value<0.001). (TIF) [file pone.0021131.s002.tif]

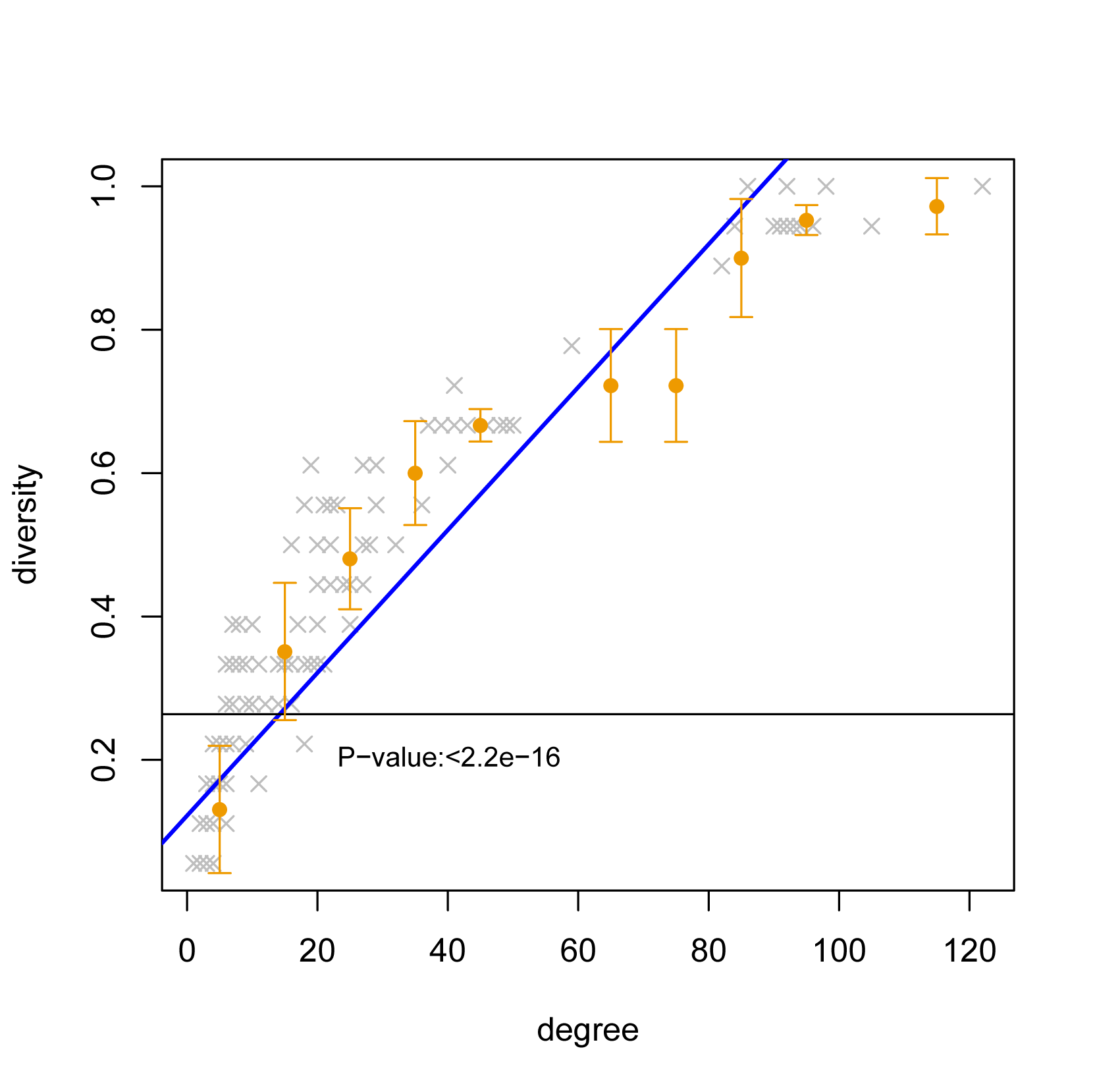

Supplement: Figure S3 — Correlation between the disease diversity and the degree within disease-related metabolic subpathways. For each subpathway in the DMSPN, the degree of subpathway and the disease diversity were calculated. Gray “×” symbols represented subpathways. Black horizontal lines are the average of value of disease diversity. Color points correspond to the binned ratio values and error bars correspond to the standard deviations of the binned ratio values. The linear regression model was used to test the trends in correlations and the significance of these trends was estimated. The result showed that there was a significant positive correlation between the disease diversity and the degree of a subpathway (P-value<2.2e–16). (TIF) [file pone.0021131.s003.tif]

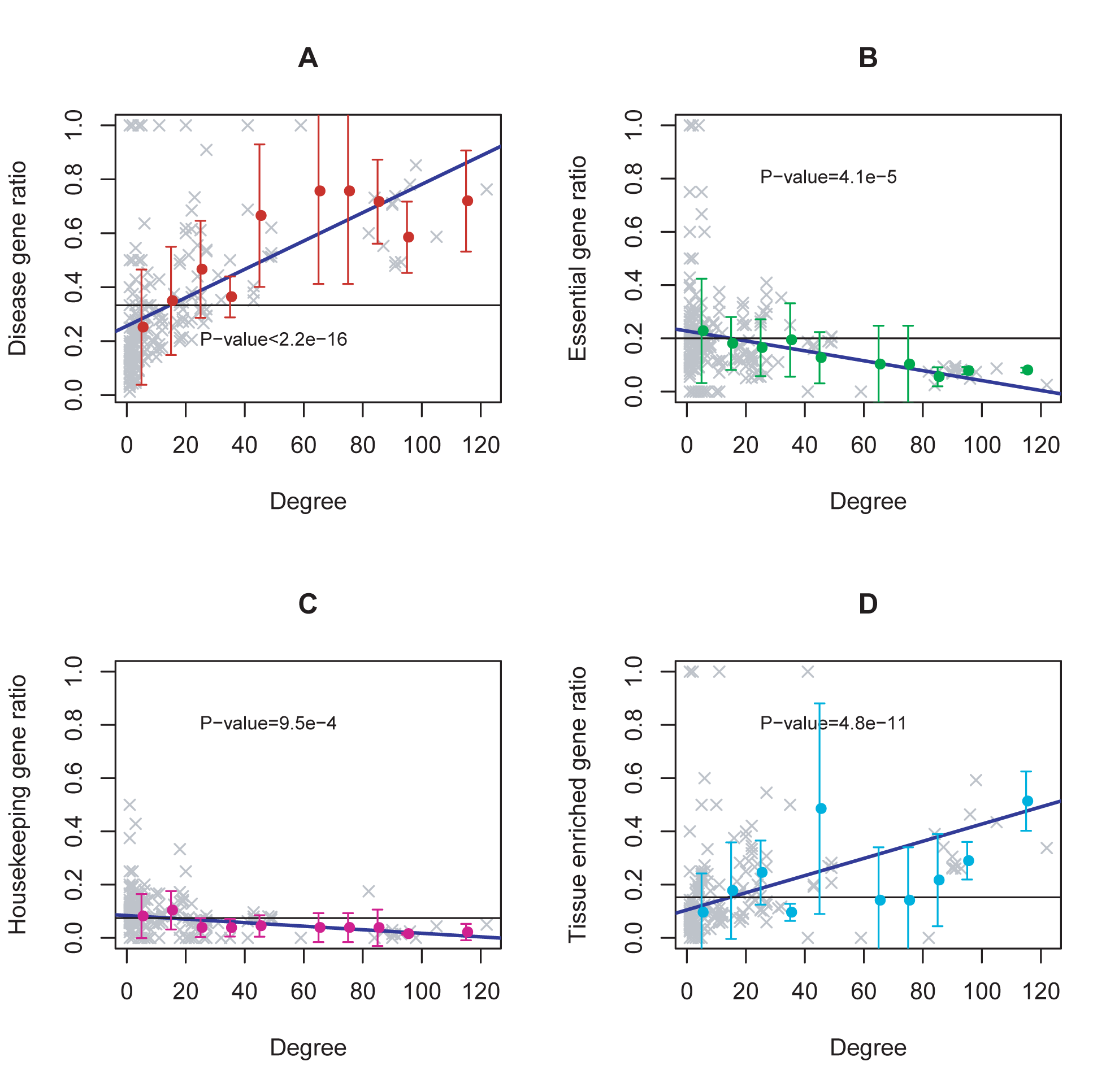

Supplement: Figure S4 — Correlations between network topology and genes within disease-related metabolic subpathways. For each subpathway in the DMSPN with k = 4, the degree of subpathway and the ratio of different types of genes within subpathway were calculated. Gray “×” symbols represented subpathways. Black horizontal lines are the average of the ratio of genes. Color points correspond to the binned ratio values and error bars correspond to the standard deviations of the binned ratio values. The linear regression model was used to test the trends in correlations and the significance of these trends was estimated. (A) The ratio of disease genes divided by all genes within the subpathways. (B) The ratio of essential genes divided by all genes in the subpathways. (C) The ratio of housekeeping genes divided by all genes in the subpathways. (D) The ratio of tissue-specific genes divided by all genes in subpathways. (TIF) [file pone.0021131.s004.tif]

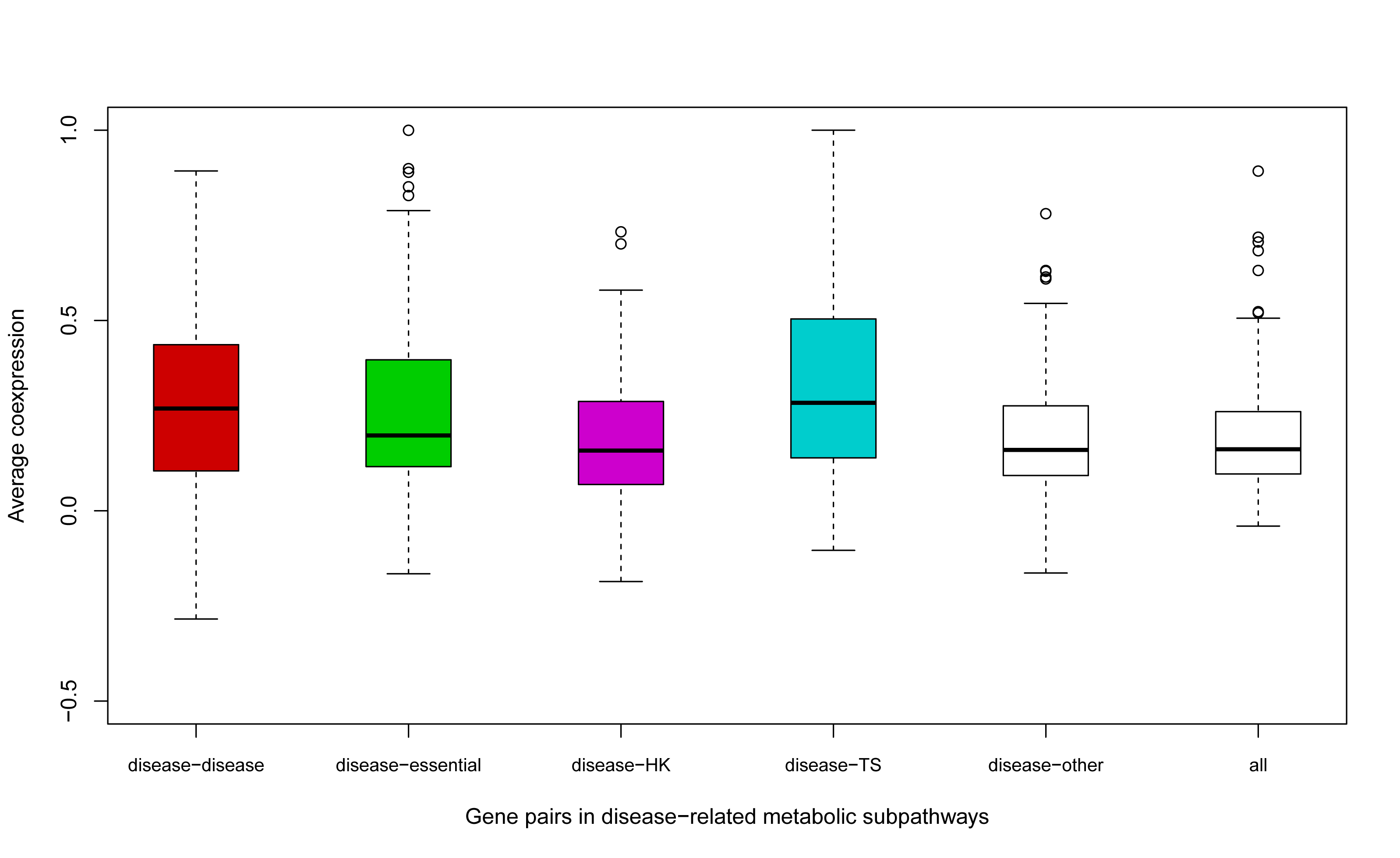

Supplement: Figure S5 — The average coexpression of gene pairs within disease-related metabolic subpathways in the DMSPN with k = 4. (TIF) [file pone.0021131.s005.tif]
