# Supplementary material for: The Implications of Relationships between Human Diseases and Metabolic Subpathways
Source: PLoS One. 2011 Jun 17;6(6):e21131. doi: 10.1371/journal.pone.0021131 (PMC3117879; doi:10.1371/journal.pone.0021131)
Supplement: Text S1 — Supplementary information text. (DOC) [file pone.0021131.s009.doc]

**Supplementary information**

**1. The Genetic Association Database (GAD)**

The GAD is the NIH supported public repository of human genetic association studies of complex diseases, which contains the complete known gene-phenotype associations and include non-mendelian common complex diseases (1). Each term of GAD is composed of broad phenotype, disease class, gene, reference, etc. We downloaded all terms of gene–phenotype relationships (N=39910) in the GAD (December 15, 2008). These terms were further parsed into 412 distinct diseases by merging broad phenotypes according to the following steps. At first, some terms have same broad phenotype names. For example, 112 “alcohol abuse” terms were merged in one category (see Supplementary information (SI) Dataset S1). This step was done first automatically and then each broad phenotype was manually tested. Secondly, because some broad phenotypes referred to the same disease were though spelled differently, we merged them in the same category. For example, the term “alcoholism” and “alcohol dependence” were merged in the disease “alcohol-dependence” (see SI Dataset S1). Thirdly, we merged some broad phenotypes according to their similarity and the physiological system they affected. For example, some terms in the disease class “infection”, whose broad phenotypes are malaria infection, echinococcosis infection, trypanosomiasis infection, etc., were merged into the disease category “parasitic infection” (see Dataset S1). Some terms in the disease class “immune”, which were relative to organ transplant such as liver, renal and bow marrow transplant and host defense, were merged into the disease category “organ transplant” (see SI Dataset S1). After the above steps, we mapped symbols of disease genes to Entrez Gene IDs, generating the list of disease–gene associations (see Dataset S2). We finally selected 15149 unique disease–gene relationships of 39910 primary terms. These associations included 412 diseases, which are grouped into 18 disease classes, and 2831 disease genes.

**2. The k-clique subpathway identification of metabolic pathways by using SubpathwayMiner software package**

In this paper, we used the “k-cliques” subpathway identification method provided by the SubpathwayMiner software package (4) to identify statistically significantly enriched disease-related subpathways. We developed the “k-cliques” subpathway identification method according to pathway structure data provided by KEGG. After inputting gene sets (in the paper, the gene sets are the given disease-related genes) and distance parameter k, the method can mine each metabolic subpathways and then identify statistically significantly enriched subpathways. The following describes the step-by-step method for identifying metabolic subpathways. First, each metabolic pathway is converted to an undirected graph with enzymes as nodes. Enzymes in a graph are connected by an edge if their corresponding reactions have a common compound. Secondly, according to parameter k, all subpathways (k-cliques) in metabolic pathways can be constructed using “k-cliques” algorithm on each above graph. The identifier of each subpathway is given with its pathway identifier plus a subpathway number (e.g. ‘path: 00010_1’). For each subpathway (k-clique), distance among all enzymes within it is no greater than the parameter k (a user-defined distance). Gene sets can then be annotated to these subpathways through assigning EC numbers for them and matching them to these subpathways. Finally, the significantly enriched subpathways can be identified using hypergeometric test.

To construct disease–metabolic subpathway network (DMSPN), we obtained 412 diseases from the GAD. These diseases corresponded to 412 disease-related gene sets, which were used to identify significantly enriched disease-related subpathways (see SI Dataset S2). When we set up k=3, 743 subpathways were generated by k-clique algorithm. Then, for each of 412 disease-related gene sets, the statistically significantly enriched subpathways were identified by the “k-cliques” subpathway identification method. As a result, we mapped 302 of the 743 subpathways to 243 among the total of 412 diseases and generated 4288 significant disease–subpathway associations (see SI Dataset S3).

We developed the function of automatically constructing disease-metabolic subpathway networks. It was integrated into the SubpathwayMiner package freely available at <http://cran.r-project.org/web/packages/SubpathwayMiner/> (Figure S1). After installing the SubpathwayMiner package in R, the generateNetwork function can be used to construct disease-metabolic subpathway network with the different distance parameter k. The following is an example command of constructing the DMSPN with k=3.

*> path<-paste(system.file(package="SubpathwayMiner"),*

*+ "/localdata/Dise2G.txt" , sep="")*

*> Dise2G<-read.table(path,header=TRUE,sep = "\t",*

*+ quote="\"", colClasses="character")*

*> DMSPN<-generateNetwork(Dise2G, k=3, pvalue=0.01)*

**3. Random network generation and analyses**

To estimate the background distribution of disease–metabolic subpathway network, we randomly shuffled the disease–gene associations, while both the number of genes that a disease is associated with and the number of diseases that a gene is associated with remain unchanged. We generated 1000 independent randomized samples. Through comparing the real DMSPN with the random network, we found that (1) the degree distribution of subpathway nodes of the actual DMSPN was significantly broader than that of random networks (P-value<10e-10); (2) the degree distribution of disease node did not display such highly significant difference (P-value=0.02); (3) the edges in the DMSPN were significantly denser than expected by chance (P-value<0.001); and (4) the size of giant component of the DMSPN was significantly smaller than expected by chance (P-value<0.001) (Figure S2).

**4. Computation of BD and BH for a disease (pathway) class in the DMSPN**

Park et al. developed D and H measures to capture the detailed interplay between the network structure and node properties when nodes in a network belong to two distinct classes (2). The D and H values are two network properties of nodes for quantifying whether nodes with similar characteristics have a tendency to link to each other. The measures have been successfully applied to modularity evaluation of protein–protein interaction networks (2) and disease networks (3). In this study, we revised the D and H measures for the one-mode networks (e.g. protein-protein interaction networks) to BD and BH measures for bipartite networks such as the disease–metabolic subpathway network (DMSPN).

When diseases in the cancer class in the DMSPN are taken as an example, we define $dn_{1}$ as the number of diseases belonging to the cancer class and $dn_{0}$ as the number of diseases not belonging to the cancer class. $dn=dn_{0}+dn_{1}$ is the total number of diseases in the DMSPN. We define $pn_{1}$ as the number of subpathways associated with cancer (means that a subpathway has a link with a disease in the cancer class) and $pn_{0}$ as the number of subpathways associated with diseases not belonging to the cancer class. $pn=pn_{0}+pn_{1}$ is the total number of subpathways in the DMASPN. If $M$ is the total number of edges in the network, $p=\frac{M}{dn\times pn}$ represent the average probability that diseases are connected with subpathways in the network. A disease may belong (1) or not belong to (0) to the cancer class. A subpathway may belong (1) or not belong (0) to the subpathway associated with cancer. Therefore, three types of edges between a disease and a subpathway exist: 1-1, 1-0 (0-1), and 0-0. $m_{11}$, $m_{10}$ and $m_{00}$ are used to represent the number for each type of edge. If each node in the network has an equal chance of associating with cancer, the expected values of $m_{11}$ and $m_{10}$ are

$$\bar{m}_{11}=\left(\begin{matrix} dn_{1} \\ 1 \end{matrix}\right)\times\left(\begin{matrix} pn_{1} \\ 1 \end{matrix}\right)\times p$$

$$\bar{m}_{10}=\left[\left(\begin{matrix} dn_{1} \\ 1 \end{matrix}\right)\times\left(\begin{matrix} pn_{0} \\ 1 \end{matrix}\right)+\left(\begin{matrix} dn_{0} \\ 1 \end{matrix}\right)\times\left(\begin{matrix} pn_{1} \\ 1 \end{matrix}\right)\right]\times p$$

Finally, the BD and BH are defined as: and .

For diseases in each disease class in the DMSPN, BD and BH can be calculated according to the above formula. BD>1 (BD<1) indicates that diseases in the disease class tend to connect more (less) densely among themselves than expected by chance. Similarly, BH>1 (BH<1) means that diseases in the disease class have more (fewer) connections to diseases in other classes than the random expectation. If BD>BH, diseases in the disease class tend to display a clustering phenomenon in the DMSPN. If BD>1 and BH<1, diseases in the disease class tend to display a clear clustering phenomenon in the DMSPN. Similarly, we are also able to calculate the BD and BH values of subpathways in a subpathway class.

**Supplementary references**

1. Becker, K.G., Barnes, K.C., Bright, T.J. and Wang, S.A. (2004) The genetic association database. *Nat Genet*, **36**, 431-432.

2. Park, J. and Barabasi, A.L. (2007) Distribution of node characteristics in complex networks. *Proc Natl Acad Sci U S A*, **104**, 17916-17920.

3. Jiang, X., Liu, B., Jiang, J., Zhao, H., Fan, M., Zhang, J., Fan, Z. and Jiang, T. (2008) Modularity in the genetic disease-phenotype network. *FEBS Lett*, **582**, 2549-2554.

4. Li, C., Li, X., Miao, Y., Wang, Q., Jiang, W., Xu, C., Li, J., Han, J., Zhang, F., Gong, B. *et al.* (2009) SubpathwayMiner: a software package for flexible identification of pathways. *Nucleic Acids Res*, **37**, e131.

5. Kharchenko, P., Church, G.M. and Vitkup, D. (2005) Expression dynamics of a cellular metabolic network. *Mol Syst Biol*, **1**, 2005 0016.

6. Ge, X., Yamamoto, S., Tsutsumi, S., Midorikawa, Y., Ihara, S., Wang, S.M. and Aburatani, H. (2005) Interpreting expression profiles of cancers by genome-wide survey of breadth of expression in normal tissues. *Genomics*, **86**, 127-141.
